# CSNK1G2-AS1 promotes metastasis, colony formation and serves as a biomarker in testicular germ cell tumor cells

**DOI:** 10.7150/jca.85640

**Published:** 2023-09-04

**Authors:** Ruixue Li, Qianyin Zhou, Guangmin Liu, Fang Zhu, Zhizhong Liu, Hao Bo, Liqing Fan

**Affiliations:** 1 NHC Key Laboratory of Human Stem Cell and Reproductive Engineering, Institute of Reproductive and Stem Cell Engineering, School of Basic Medical Science, Central South University, Changsha, 410078, People's Republic of China.; 2 Hunan Cancer Hospital, the Affiliated Cancer Hospital of Xiangya School of Medicine, Central South University, Changsha, 410078, People's Republic of China.; 3 Clinical Research Center for Reproduction and Genetics in Hunan Province, Reproductive and Genetic Hospital of CITIC-Xiangya, Changsha, 410078, People's Republic of China.

**Keywords:** Testicular germ cell tumors (TGCTs), long noncoding RNA (lncRNA), metastasis, EMT, AKT

## Abstract

**Background/Aim:** Some long non-coding RNAs (lncRNAs) have been found to significantly participate in the progression of TGCTs. In comparison to the normal testis, the TGCT tissues showed significantly decreased *CSNK1G2-AS1* expression, however, its effect on TGCTs and its mechanism are still unclear. The aim of this study is to investigate the effect of *CSNK1G2-AS1* on TGCTs and explore the mechanism underlying its effect on TGCTs.

**Materials and Methods:** In this study, to evaluate the expression of *CSNK1G2-AS1* in tissue samples from TGCTs, the UCSC and GEPIA databases were applied and qRT-PCR was conducted. The Kaplan-Meier Plotter was applied to analyze the correlation between *CSNK1G2-AS1* methylation levels and the prognosis of TGCTs patients. The assays of MTS, clone formation, transwell, and flow cytometry were performed to investigate the effect of *CSNK1G2-AS1* overexpression on the proliferation, metastasis, and apoptosis of TGCT cells, respectively. Finally, western blotting was conducted to determine the expressions of the proteins associated with EMT and AKT.

**Results:** Our study first found that, compared to the normal testis, TGCTs tissue showed significantly decreased *CSNK1G2-AS1* expression, and hypomethylation of *CSNK1G2-AS1* was significantly correlated with a better prognosis with TGCTs patients. *In vitro*, we found that overexpression of *CSNK1G2-AS1* dramatically promoted the clone formation, invasion, and migration of TGCT cells, but inhibited apoptosis. And *CSNK1G2-AS1* overexpression significantly decreased the expression of EMT-associated proteins ZO-1 but increased the expression and phosphorylation of AKT.

**Conclusions:** *CSNK1G2-AS1* may play an essential role in the pathogenesis and metastasis of TGCTs through the EMT- and AKT-mediated signal pathways.

## Introduction

Testicular germ cell tumors (TGCTs) are the most common solid tumors in adolescent and male young adults 1. Over the past few decades, TGCTs have continued to increase in the majority of the population 2. By 2030, it is estimated that there will be 65,827 new cases of testicular cancer worldwide (10,561 more cases than in 2012) 3. TGCTs are roughly classified into seminomas and non-seminomas, of which non-seminomas include undifferentiated embryonic carcinoma or differentiated teratoma, yolk sac tumor, and choriocarcinoma 4, 5. Mixed TGCTs are common and may appear in any form. The clinical management of TGCTs can be effectively treated by monitoring, surgery, chemotherapy, radiotherapy, or combining them 6. Although the 5-year relative survival rates for seminoma and non-seminoma patients are above 90% 7, there is still a risk existed. According to statistics, approximately 15% to 20% of patients with the disseminated disease will relapse, especially in the late stage (2 years after remission), with a poor prognosis 8. Metastasis and cisplatin resistance are presently the two major causes of death in most TGCT patients 9. The Serum tumor markers, such as α-fetoprotein (AFP), β-subunit of chorionic gonadotropin (β-hCG), and lactate dehydrogenase (LDH), can be used for the diagnosis of testicular tumors and the monitoring of disease recurrence 10. However, false positive or negative results can always arise 11, 12. The genes driving the tumorigenesis or metastasis of TGCTs have not been identified so far, and these genes may be potential therapeutic targets 13. Despite recent efforts to identify other susceptible genetic/epigenetic lesions, no effective molecular markers are available for screening, diagnosis, or prognosis 14. Therefore, identifying the potential mechanism underlying TGCTs and novel biomarkers for target therapies are great importance and urgently needed.

Recently, increasing amounts of studies have shown that the epigenetic regulations are closely connected to the pathogenesis of TGCTs 15. LncRNAs are considered a class of nonprotein-coding transcripts with a minimum length of 200 bases. In recent years, lncRNAs have been increasingly investigated in various human diseases, like cancer 16-18. Through regulating miRNAs, chromatin remodelling, and histone modification, lncRNAs significantly participated in the regulation of cell growth, apoptosis, metastasis, and angiogenesis in malignant tumors 19. Alterations of lncRNA expression might be closely associated with the tumorigenesis of TGCTs 20, 21. For example, the HOXA transcript at the distal tip (HOTTIP) was a noncoding RNA with 3764 nucleotides, and significantly participated in the promotion of cell proliferation through competitive binding of miR-128-3p in testicular embryonic carcinoma 22. LncRNA Narcolepsy candidate-region 1 C (NLC1-C) was found to be down-regulated in testicular embryonal carcinoma cells, and exerted a tumor-promoting role in testicular cancer 23. Therefore, understanding the function of lncRNA molecules in tumor is conducive to identify new diagnostic biomarkers or therapeutic targets for TGCTs 20, 21.

LncRNA casein kinase 1 gamma 2 antisense RNA 1 (*CSNK1G2-AS1*), also known as chromosome 19 open reading frame 34 (*C19orf34*), is an RNA with 1,313 kb in 19p13 and highly expressed in the testis. *CSNK1G2-AS1* is the antisense RNA of *CSNK1G2*, located on the complementary sequence of intron 1 of *CSNK1G2*. *CSNK1G2* is a major negative regulator of necroptosis, and the *CSNK1G2* knockout mice showed premature aging of their testis 24. In our present study, we analyzed the expression of *CSNK1G2-AS1* in TGCTs and its relationship with the prognosis of TGCT patients using the Gene Expression Profiling Interactive Analysis (GEPIA) database 25 (http://gepia.cancer-pku.cn/) and University of California Santa Cruz (UCSC) Xena database, respectively. We found that compared to the normal testis tissues, *CSNK1G2-AS1* was significantly down-regulated in TGCT tissues. However, its role in TGCTs and the mechanism underlying the role are still unclear.

In our study, we reported for the first time that *CSNK1G2-AS1* was down-regulated in TGCTs and the hypomethylation of *CSNK1G2-AS1* was significantly associated with a better prognosis in patients with TGCTs. We hypothesized that *CSNK1G2-AS1* is involved in tumorigenesis and progression of TGCTs. Then we confirmed for the first time the expression of *CSNK1G2-AS*1 in testicular germ cell tumor tissues and cells. The purpose of this study was to investigate the function of *CSNK1G2-AS1* in the development of TGCTs, and identify additional therapeutic targets and molecular markers for the clinical treatment and diagnosis of TGCTs.

## Materials and methods

### Collection of patient testicular tissue samples

Eleven TGCT tissue samples and ten adjacent normal testicular tissue samples were collected from the Department of Urology, the Affiliated Cancer Hospital, Xiangya School of Medicine, Central South University for qRT-PCR detection. This study was approved by the Ethics Committee of Central South University (2021KYKS-46). The informed consent was signed by each patient enrolled in this study. All tissues were confirmed by histopathological examination from the Pathology Department of Hunan Cancer Hospital. Fresh tissues were collected and frozen in liquid nitrogen for storage at -196°C.

### Analysis of *CSNK1G2-AS1* expression in TGCTs and the total testicular cell population

The expression of *CSNK1G2-AS1* in various organs was analyzed using the UCSC database 26 (http://genome.ucsc.edu/). The expression of *CSNK1G2-AS1* in TGCTs and normal testicular tissues was evaluated using GEPIA database 25 (http://gepia.cancer-pku.cn/). The RNA sequencing data of 13 tissue samples from TGCT patients and 4 paraneoplastic tissue samples (in our previous study 27) were also applied for the analysis of the *CSNK1G2-AS1* expression. To investigate the expression level of *CSNK1G2-AS1* in the total testicular cell population, we retrieved the single cell RNA sequencing data of Human Testis Cell Atlas from the Gene Expression Omnibus (GEO) database (accession number: GSE120508) (https://humantestisatlas.shinyapps.io/humantestisatlas1/) 28.

### Evaluation of *CSNK1G2-AS1* methylation in TGCTs and prognosis of TGCT patients

The CpG islands around *CSNK1G2-AS1* were profiled by the UCSC Genome online tool (https://genome.ucsc.edu/) 26, The data of *CSNK1G2-AS1* methylation and prognosis of patients with TGCTs were downloaded from the UCSC Xena database (https://xena.ucsc.edu/) 29. The relationship of the *CSNK1G2-AS1* methylation with the prognosis of patients with TGCTs was analyzed using the Kaplan-Meier Plotter (https://kmplot.com/analysis/) 30. Progression-free survival (PFS), defined as the time it takes after treatment to disease progression or death 31, 32. Disease-free survival (DFS), defined as the time after treatment during which no disease is found.

### RNA isolation and qRT-PCR detection

According to the manufacturer's instructions, total RNA was extracted from tissues or cells using TRIzol reagent (Invitrogen, Waltham, MA, USA). The quality and purity of each RNA sample were measured by Agilent2100 (Agilent, Wilmington, DE, USA). The cDNA was synthesized from 1 µg of extracted RNA using the Transcriptor First Strand cDNA Synthesis Kit (Roche, Basel, Switzerland). The cDNA product was diluted fivefold with enzyme-free water, and then the LightCycler 480 SYBR Green I Master (Roche) was operated as instructed by the manufacturer. *β-actin* was used as the internal control and all experiments were repeated three times indenpently. Amplification was carried out using the following thermocycling conditions: initial denaturation at 95°C for 5 min, followed by 45 cycles of 95°C for 10 s and 60°C for 10 s, and final extension at 72°C for 10 s. The LightCycler 480 software (Roche) was used to analyze threshold cycle (CT) values, and the 2^-∆∆CT^ method was used to evaluate the relative gene expression 33. The sequences of the primers for qRT-PCR detection were listed below: *CSNK1G2-AS1* Forward: GACAGCCGGCCTCTGAGAAC, Reverse: TCCACTTTGCTTTTGTTGATGGCGT; *β-actin* Forward: TTCCAGCCTTCCTTCCTGGG, Reverse: TTGCGCTCAGGAGGAGCAAT.

### Cell culture and cell transfection

The NCCIT human malignant teratoma cells were purchased from the American Type Culture Collection (ATCC, VA, USA). The TCam-2 human testicular seminoma cells were obtained from Dr. Yuxin Tang 34, 35. NCCIT and TCam-2 cells were cultured in RPMI 1640 (GIBCO, Grand Island, NY, USA) or Dulbecco's Modified Eagle's Modified (DMEM, GIBCO) medium 36-38 supplemented with 10% fetal bovine serum (FBS, GIBCO), 100 U/mL penicillin and 100 μg/mL streptomycin (GIBCO) respectively, in an incubator containing 5% CO_2_ at 37°C. The cells at logarithmic phage were collected and transferred to a 6-well plate (5.0 × 10^5^ cells/well) and transfected at 70% confluence. As previously described 27, cells were divided into two groups: the cells transfected with 2.5 µg of the pcDNA3.1(+) vector and the cells transfected with 2.5 µg of the *CSNK1G2-AS1* pcDNA3.1(+) vector. Cell transfection was performed using lipofectamine 3000 (Invitrogen) according to the manufacturer's instructions. Cells were collected 48 h after transfection for subsequent experiments. The cDNA of *CSNK1G2-AS1* was PCR-amplified and subcloned into the HindIII and XhoI sites of the pcDNA3.1(+) vector. The *CSNK1G2-AS1* overexpression plasmid was designed and synthesized by Bochu Biotechnology Co., Ltd (Changsha, China).

### MTS assay

The 3-(4,5-dimethylthiazol-2-yl)-5-(3-carboxymethoxyphenyl)-2-(4-sulfophenyl)-2H-tetrazolium (MTS) reagent (Promega) was used to detect cell proliferation. TCam-2 and NCCIT cells were cultured in a 96-well plate with 5 × 10^3^ cells/mL (200 μL/well) for 6 h as day 0 and then transfected with indicated plasmids at 24, 48, 72, 96, and 120 h (day 1, day 2, day 3, day 4, day 5, respectively). Then 20 μL MTS was added and incubated for 4 h at 37°C. The absorbance at 492 nm was determined by the enzyme immunoassay analyzer (Thermo Fisher Scientific, Waltham, MA, USA). Each experiment was repeated three times.

### Plate colony formation assay

The transfected cells were inoculated into a 6-well plate at a density of 400 cells/well and cultured in an incubator at 37°C and 5% CO_2_ for eight days until most single cells could be observed under a microscope to become clones containing 50 cells. The cells were then fixed with 4% paraformaldehyde for 30 min and stained with 0.1% crystal violet (Sigma, St. Louis, MO, USA) for 15 min. After dyeing, a photo was taken to calculate the number of clones using Adobe Photoshop CS5. Data from three independent experiments were expressed as mean ± standard deviation (S.D.).

### Flow cytometric cell apoptosis assay

After 48 h of transfection, cells were digested into single cell with 0.05% trypsin (GIBCO), washed with pre-cooled PBS (Gibco) three times, and treated with an Annexin V FITC Apoptosis Kit (BD Pharmingen; San Diego, CA, USA) according to the manufacturer's instructions. The Accuri C6 flow cytometer (BD) (FACScan) was employed for detection of apoptosis. Finally, FlowJo VX 10 (Tree Star; Ashland, OR, USA) was applied to analyze the apoptosis results. Each experiment was performed in triplicate.

### Transwell cell migration and invasion assays

Transwell migration and invasion assays were performed using 8.0 μm Transwell Permeable

Support (353097) (Corning Inc., Corning, NY, USA). TCam-2 and NCCIT cells were digested into single cell and resuspended with serum-free substrate after 48 h of transfection. Cells were diluted to a concentration of 100,000/mL, 200 μL cells were added to the upper chamber which was precoated with Matrigel Matrix (BD) 27, and 800 μL 15% FBS-contained medium was added to the lower chamber. The 24-well plates were placed in an incubator at 37°C and with 5% CO_2_ for 48 h. The chamber was then washed twice with PBS, fixed with 4% paraformaldehyde on the upper and lower walls for 30 min. After another washing with PBS, the fixed cells were stained with 0.1% crystal violet (Sigma) for 15 min. Then the cells on the upper surface of the chamber that did not pass through the chamber were rinsed with clean water and gently removed with a sterile cotton swab. Finally, cells were observed and photographed under an inverted microscope and counted in five randomly selected fields. The experiment described above was repeated three times.

### Western blotting

Cells were collected and lysed using radioimmunoprecipitation assay (RIPA) lysis buffer (CoWin Biosciences, Beijing, China) according to the manufacturer's instructions to obtain the protein. The Bicinchoninic Acid (BCA) Protein Quantification Kit (Thermo Fisher Scientific) was used for protein quantification, and the samples were adjusted to the same concentration. After mixing with 5× loading buffer (CoWin Biosciences), the protein samples were boiled at at 100°C for 8 min, and then the protein samples were separated by 10% sodium dodecyl sulfate-polyacrylamide gel electrophoresis gels (SDS-PAGE) and transferred to the polyvinylidene fluoride (PVDF) membrane (Millipore, Billerica, MA, USA). After blocking with 5% fatty-free milk for 1 h, the corresponding primary antibodies were incubated with the PVDF membrane overnight at 4°C. Next, After 3-time washing using Tris Buffered Saline with Tween-20 (TBST), the PVDF membrane was incubated with the secondary antibody at 37°C for 1 h. After 3-time washing with TBST, the protein bands were visualized by fluorography using an enhanced chemiluminescence system (Millipore). Primary antibodies used in this study are as follows: anti-glyceraldehyde-3-phosphate dehydrogenase antibody (GAPDH) (CW0100) (1:1,000 dilution; CoWin Biosciences), anti-zonula occludens-1 (ZO-1) antibody (#8193) (1:1,000 dilution; Cell Signaling Technology), anti-AKT antibody (10176-2-AP) (1:1,000 dilution; ProteinTech), and anti-Phospho-AKT (Ser473) (P-AKT) antibody (66444-1-Ig) (1:1,000 dilution; ProteinTech). Secondary antibodies used are as follows: goat anti-mouse IgG (CW0102) (1:2,000 dilution; CoWin Biosciences) or goat anti-rabbit IgG (CW0103) (1:2,000 dilution; CoWin Biosciences). GAPDH was used as an internal reference. Densitometric analysis of the bands was performed using ImageJ 1.52a software (National Institutes of Health, Bethesda, MD, USA) for protein quantification.

### Statistical analysis

The data were represented as means ± standard deviation (S.D.) and each experiment was repeated at least three times independently. The results were analyzed using GraphPad Prism software (Version 5.0; La Jolla, CA, USA). The Student's t test was used to calculate the difference between the two groups. Significant differences between multiple data sets were calculated using one-way analysis of variance (ANOVA). P < 0.05 was deemed statistically significant. PFS and DFS were calculated using a log-rank test.

## Results

In the present study, we tried to explore the function of *CSNK1G2-AS1* in TGCTs based on a series of bioinformatic analysis and *in vitro* experiments. We found that *CSNK1G2-AS1* had a lower expression in TGCTs and that hypomethylation of *CSNK1G2-AS1* was significantly associated with a better prognosis of patients with TGCTs. Further analysis showed that *CSNK1G2-AS1* could promote the migration and invasion of TGCT cells and regulate the expression of AKT and the proteins associated with EMT.

### Decreased *CSNK1G2-AS1* expression in TGCT tissues and significant association between the *CSNK1G2-AS1* hypomethylation and the better prognosis

**Figure 1:**
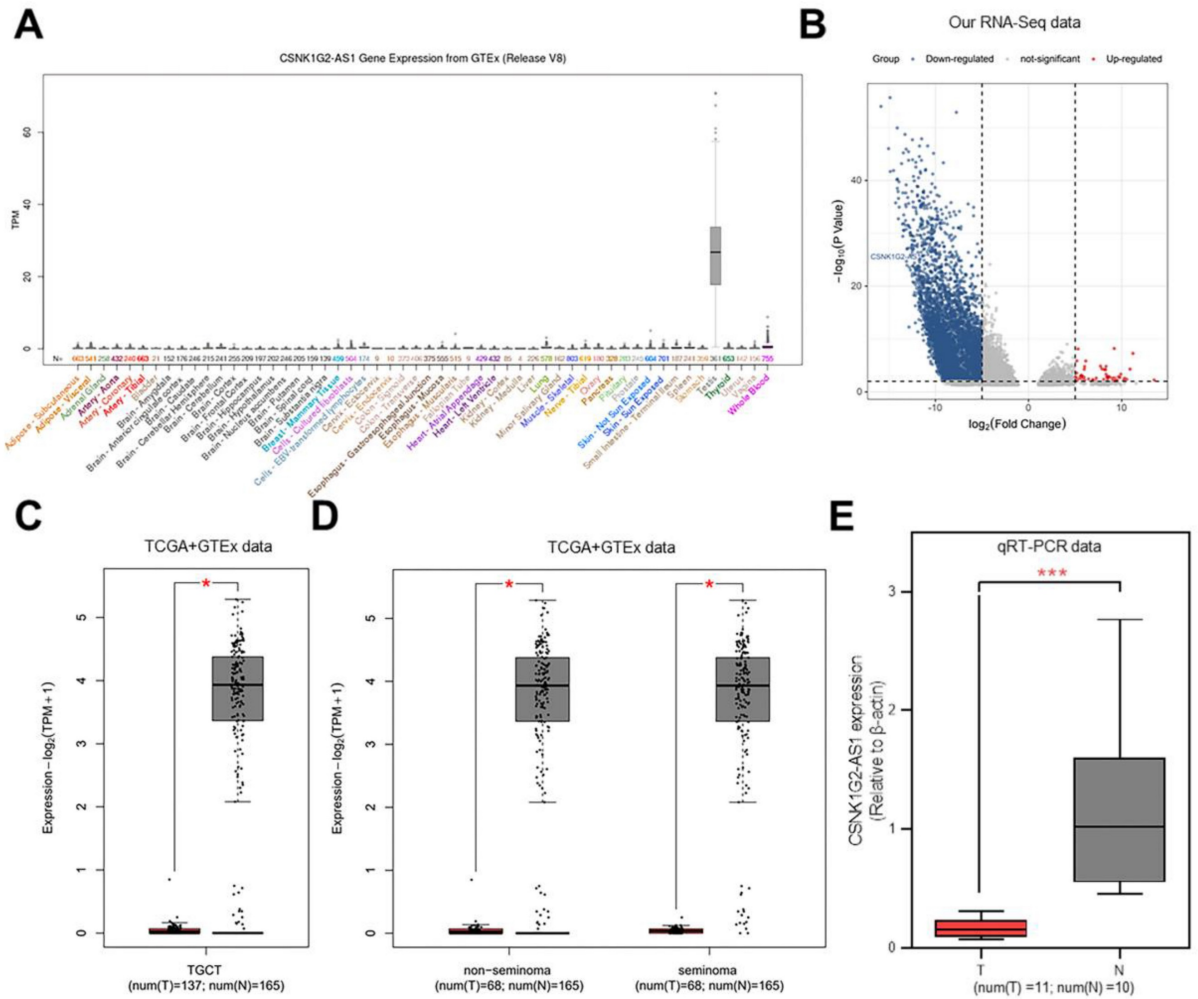
** Expression of *CSNK1G2*-*AS1* in tumor tissues.** (A) The UCSC database analysis showed that *CSNK1G2-AS1* is highly expressed in testis. (B) Representative volcano plots showing that *CSNK1G2-AS1* is significantly down-regulated in TGCT tissues. (C) Representive results from the analysis of GEPIA database showed that the expression of *CSNK1G2-AS1* in TGCT tissues was significantly lower than that of normal testis. TPM = transcribed per million. (D) Differential expression of *CSNK1G2-AS1* in seminomas and non-seminomas versus normal testis from the GEPIA database. TPM = transcribed per million. (E) qRT-PCR was used to verify the expression of *CSNK1G2-AS1* in TGCTs and normal testis. 'T' means TGCT samples, 'N' means normal testis. *P < 0.05, *** *P* < 0.001.

The levels of *CSNK1G2-AS1* expression in different tissues and organs were compared using UCSC database 26. The results showed significantly increased expression of *CSNK1G2-AS1* in the testis (Fig. 1A). In our previous study, to explore the function of lncRNAs in TGCTs, we conducted RNA sequencing and constructed lncRNA expression profiles using 13 TGCT tissues and 4 paraneoplastic tissues 27, and observed that *CSNK1G2-AS1* was significantly down-regulated in TGCTs (Fig. 1B). To verify the reliability of differential expression of* CSNK1G2-AS1,* we analyzed its expression in TGCTs using the GEPIA database 25. The results showed that *CSNK1G2-AS1* was distinctly down-regulated in different types of TGCTs (seminoma and non-seminoma) (Fig. 1C-D). Subsequently, ten adjacent normal testicular tissue samples and eleven TGCT samples were employed, and the decreased *CSNK1G2-AS1* expression in TGCTs was confirmed using qRT-PCR detection (Fig. 1E). These results revealed that compared with the normal testis, TGCT tissues exhibited significantly decreased *CSNK1G2-AS1* expression.

**Figure 2:**
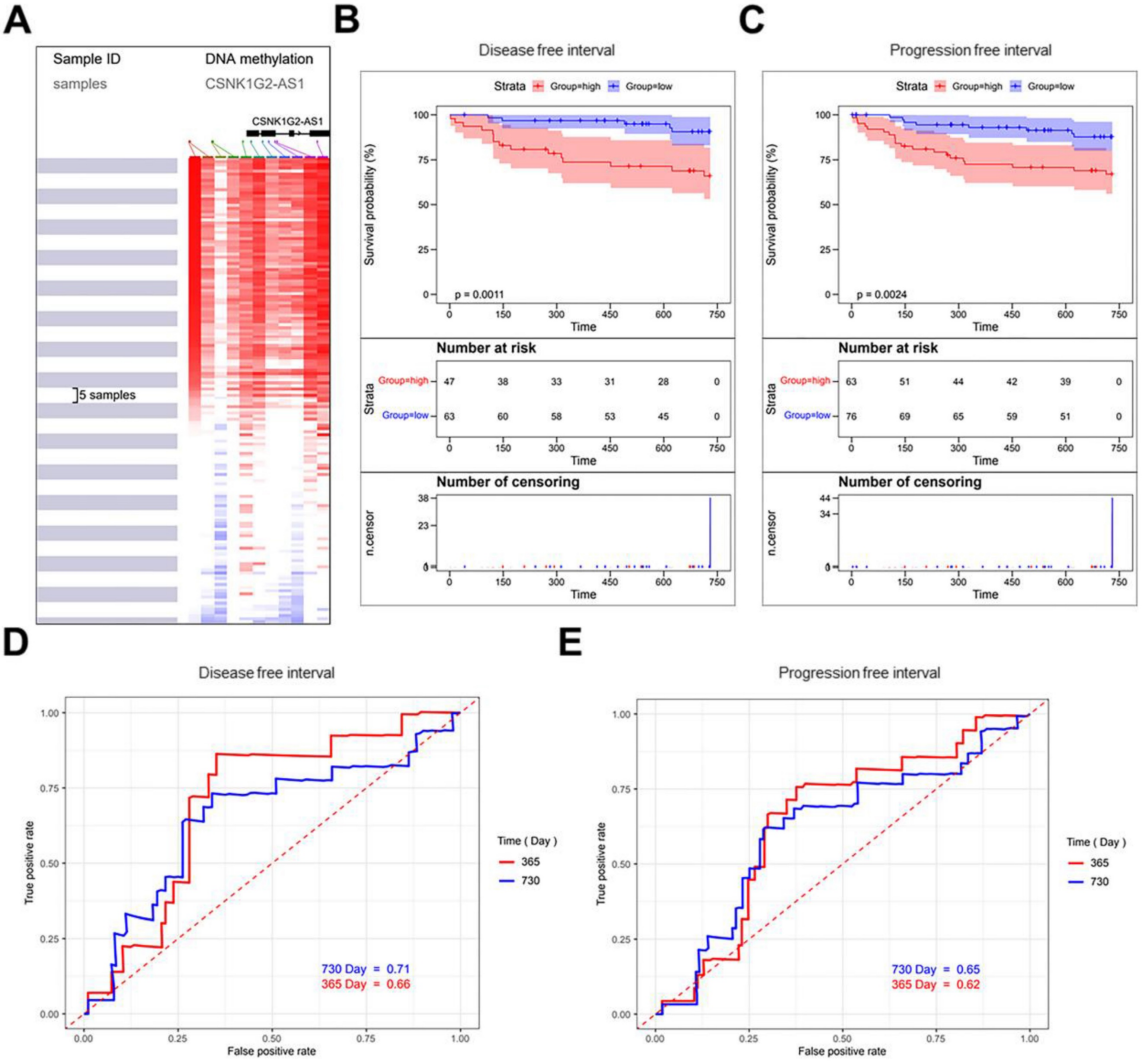
** Methylation level of *CSNK1G2-AS1* in TGCTs and the prognosis of patients with TGCTs.** (A) The CpG islands around *CSNK1G2-AS1* were profiled using the UCSC Xena online tool. (B-C) TGCT data from the TCGA TGCT cohort were used to analyze the correlation between the level of *CSNK1G2-AS1* methylation and the prognosis of patients with TGCTs. (D) Representative ROC curves showing the predictive sensitivity and specificity of the DFS at 1 and 2 years. (E) Representative ROC curves showing the predictive sensitivity and specificity of PFS at 1 and 2 years.

The CpG islands around *CSNK1G2-AS1* were profiled using the UCSC Xena online tool (Fig. 2A), and there are 156 samples in TCGA Testicular Cancer (TGCT) (17 datasets) were selected. The correlation analysis between the methylation levels of *CSNK1G2-AS1* and the prognosis of TGCT patients showed that patients with hypermethylation of *CSNK1G2-AS1* had significantly lower DFS (Fig. 2B) and PFS (Fig. 2C) than patients with hypomethylation. Finally, we plotted the time-dependent receiver operating characteristic (ROC) curves of DFS and PFS. The Area Under Curves (AUC) at 1 and 2 years of DFS were 0.66 and 0.71, respectively (Fig. 2D). The AUC at 1 and 2 years of PFS were 0.62 and 0.65, respectively (Fig. 2E). Therefore, these results suggested that the low methylation level of *CSNK1G2-AS1* may be a potential prognostic marker for a good prognosis in TGCT patients.

### Overexpression of *CSNK1G2-AS1* increased clone formation but did not affect cell proliferation

**Figure 3:**
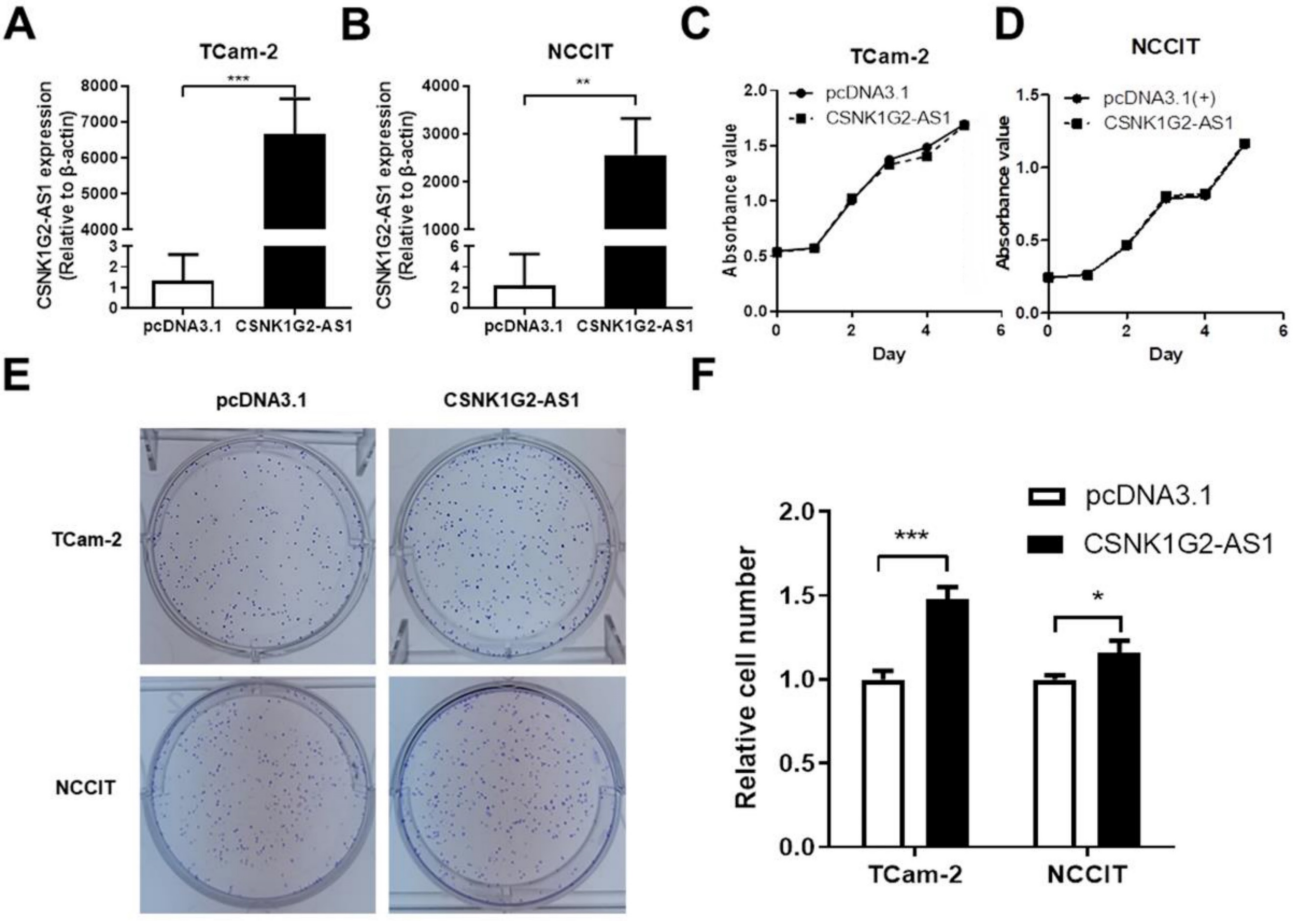
***CSNK1G2-AS1* promotes the clonal formation of TGCT cells.** (A-B) The overexpression of *CSNK1G2-AS1* in TCam-2 and NCCIT cells was verified by qRT-PCR. (C-D) MTS showed that *CSNK1G2-AS1* overexpression did not affect TCam-2 and NCCIT proliferation. (E) Colony formation assays showed that *CSNK1G2-AS1* overexpression promotes colony formation activity of TGCT cells. (F) Overexpression of *CSNK1G2-AS1* increased the relative number of TGCT cell clones. Data represent the mean ± S.D. *P < 0.05, **P < 0.01, ***P < 0.001.

Because *CSNK1G2-AS1* expression was decreased in TGCT tissues, we first tested whether overexpression of* CSNK1G2-AS1* had an effect on TGCT cell proliferation. The effect of *CSNK1G2-AS1* overexpression in TCam-2 and NCCIT cells was determined by qRT-PCR (Fig. 3A-B), then the proliferation was evaluated by the MTS assay. According to the MTS assay, we found that no significant difference was observed between the cells with* CSNK1G2-AS1* overexpression or not (Fig. 3C-D). However, significantly high clonal formation was observed in the cells with *CSNK1G2-AS1* overexpression compared to the negative control (P < 0.05, Fig. 3E-F).

### *CSNK1G2-AS1* overexpression inhibited the apoptosis of TGCT cells

**Figure 4:**
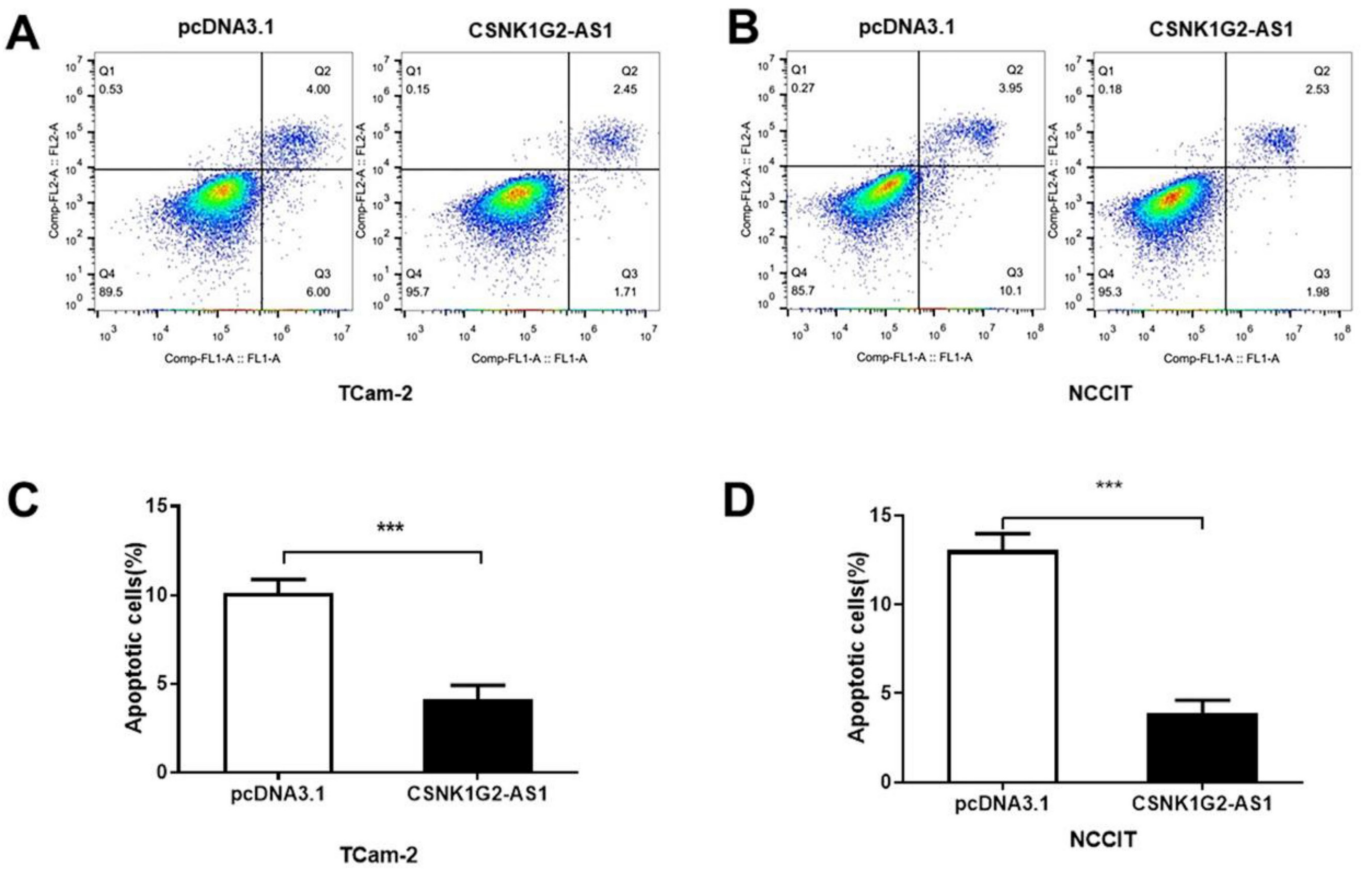
** Overexpression of *CSNK1G2-AS1* inhibits TGCT cell apoptosis.** (A-B) Flow cytometry was used to detect cell apoptosis of TGCT cells overexpressed with *CSNK1G2-AS1* and pcDNA3.1 (+). (C-D) Compared to the pcDNA3.1 (+) group, the apoptosis rate of TGCT cells overexpressing *CSNK1G2-AS1* was significantly reduced. *** *P* < 0.001.

To elucidate the influence of *CSNK1G2-AS1* on the regulation of TGCT cell apoptosis, Annexin V/PI staining and flow cytometry were performed. As shown in Figure 4, the percentages of early and late apoptotic TCam-2 and NCCIT cells were obviously reduced by *CSNK1G2-AS1* overexpression compared to the negative control (P < 0.05, Fig. 4A-D). These results showed that overexpression of *CSNK1G2-AS1* could inhibit the apoptosis of TGCT cells.

### *CSNK1G2-AS1* positively regulated the migration and invasion of TGCT cells

**Figure 5:**
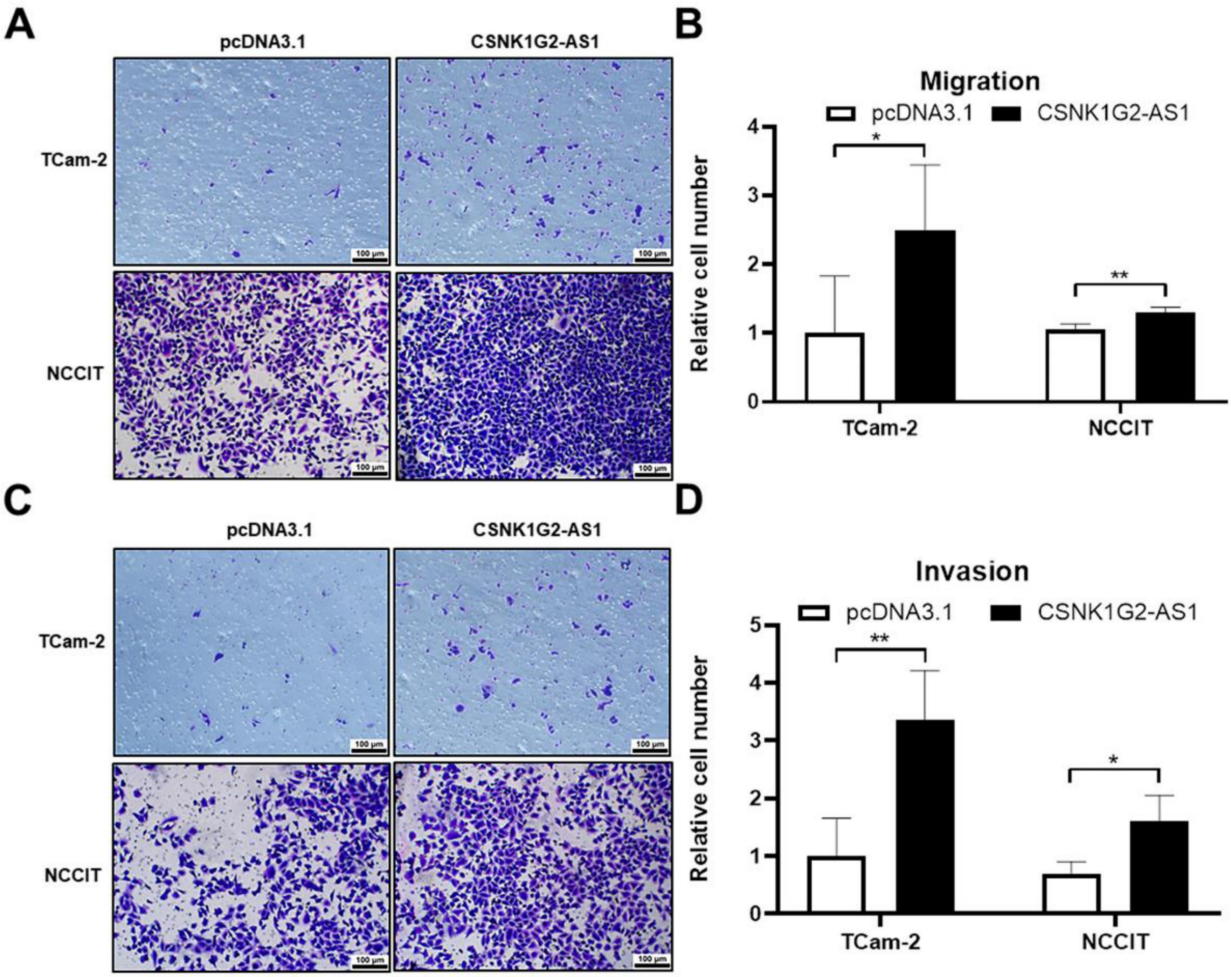
***CSNK1G2-AS1* promotes metastasis and invasion of TGCT cells.** (A) Transwell assay found that overexpression of *CSNK1G2-AS1* promotes TGCT cells metastasis compared to pcDNA3.1 (+) group. (B) The relative number of migrating cells in the overexpression of *CSNK1G2-AS1* group was increased. (C) The transwell assay found that *CSNK1G2-AS1* overexpression promoted invasion of TGCT cells compared to negative control. (D) The relative number of invasive TGCT cells with* CSNK1G2-AS1* overexpression was increased. *P < 0.05, **P < 0.01. Scale bar = 100 μm.

After orchiectomy, the probability of recurrence and metastasis in patients with stage I seminoma and patients with non-seminoma was 15-20% and ~30%, according to the European Guidelines for Urological Testicular Cancer 39. Therefore, to explore the relationship between *CSNK1G2-AS1* overexpression and the migrative and invasive abilities of TGCT cells, we performed transwell assays. We found that 48 h after overexpression, the TGCT cells exhibited significantly enhanced migrative (Fig. 5A-B) and invasive (Fig. 5C-D) abilities compared to negative control (P < 0.05). These results indicated that *CSNK1G2-AS1* plays a promoting role in the migration and invasion of TGCT cells, which warrants further investigation.

### *CSNK1G2-AS1* promoted the development of TGCT cells by affecting the signaling pathways mediated by EMT and AKT

**Figure 6:**
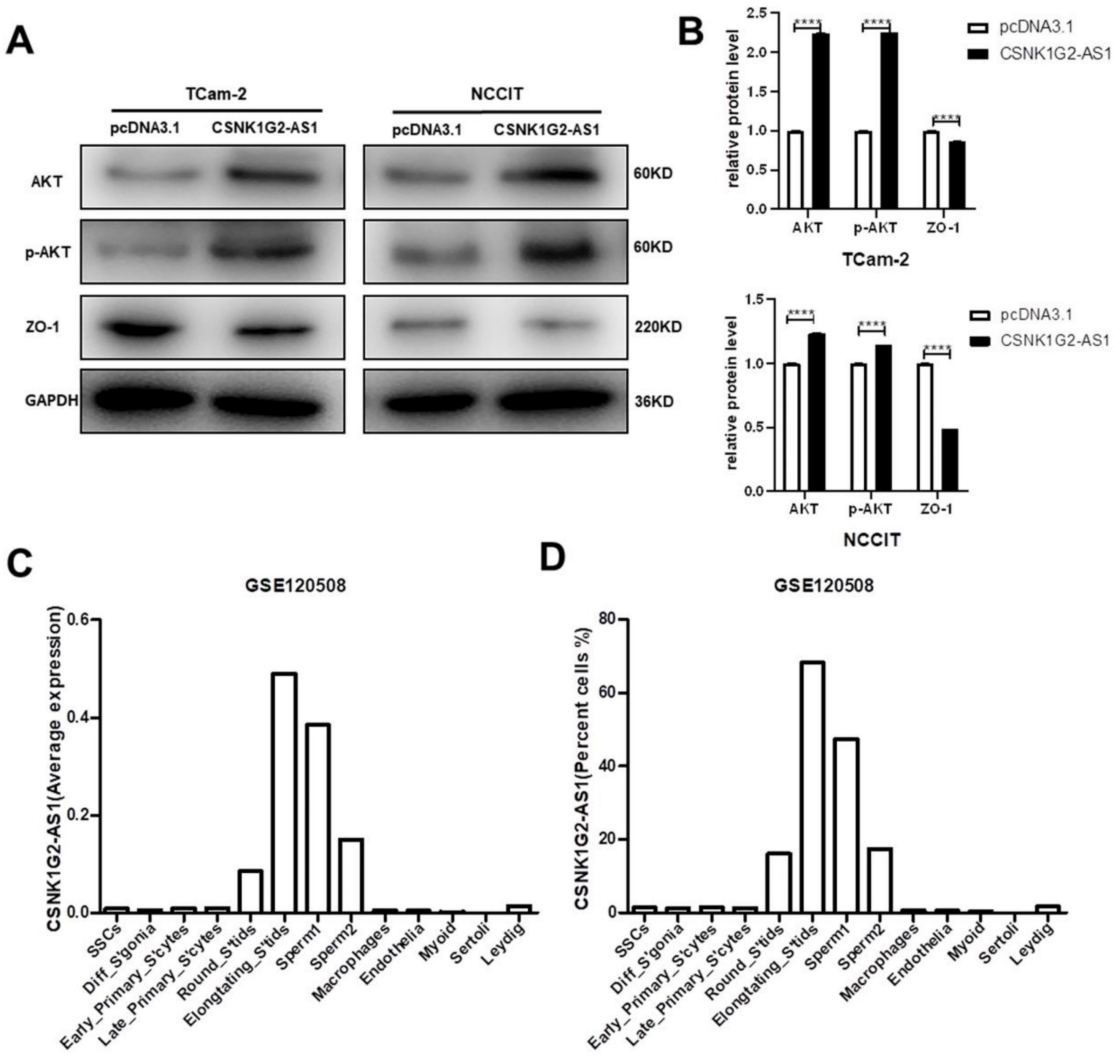
** Effects of *CSNK1G2-AS1* on EMT, AKT signaling pathway related proteins, and expression levels of *CSNK1G2-AS1* in the total testicular cell population.** (A) Western blot assays detected the expression levels of AKT, p-AKT and ZO-1 in TCam-2 and NCCIT cells. (B) Relative expression levels of AKT, p-AKT and ZO-1 in TCam-2 and NCCIT cells. GAPDH was used as a loading control. (C-D) Average expression and cell percentage of *CSNK1G2-AS1* in the human testis from the Gene Expression Omnibus (GEO) database (accession number: GSE120508). ****P < 0.0001.

Epithelial-mesenchymal transformation (EMT) is a biological process in which epithelial cells are transformed into phenotypic mesenchymal cells by a specific procedure 40. It plays an important role in cancer metastasis. To find out whether *CSNK1G2-AS1* could regulate the metastasis and invasion of TGCT cells through EMT signaling pathway, the proteins associated with EMT were detected by Western blot. We found that in TGCT cells, *CSNK1G2-AS1* overexpression dramatically enhanced the expression of the EMT-related protein ZO-1 (Fig. 6A-B). Due to the involvement of the AKT-related signaling pathway in the progression of TGCTs by regulating cell proliferation and migration 41, 42, we also detected the proteins related to the AKT signaling pathway and found that *CSNK1G2-AS1* overexpression also significantly enhanced the expression and phosphorylation of AKT. These results indicated that *CSNK1G2-AS1* could promote the development of TGCT cells by influencing the signaling pathways related to EMT and AKT (Fig. 6A-B).

### Expression of *CSNK1G2-AS1* in the total testicular cell population

The results verified by *in vitro* experiments appear to be inconsistent with bioinformatic analysis. The two main cell groups in normal testis include germ cells and somatic cells. The germ cells include a few undifferentiated spermatogonia and differentiated spermatogonia, and a large number of spermatocytes and spermatids, especially meiotic divisions and late spermatids 43, 44. The average expression (Fig. 6C) and cell percentage (Fig. 6D) of *CSNK1G2-AS1* in germ cells suggested that in the spermatogenesis process, *CSNK1G2-AS1* highly expressed in later stages of spermatogenesis (round spermatids, elongated spermatids, and sperm), but low in the proliferative stage of spermatogonial stem cells and meiophase.

## Discussion

Recently, the imbalance of lncRNAs in various cancers has attracted much more attentions. Many studies have shown that lncRNAs play a crucial role in the physiological and pathological changes of cancers 45, 46. Moreover, some studies have also shown that lncRNAs are significantly associated with the pathogenesis and development of TGCTs. For example, the removal of SPRY4-IT1 lncRNA can inhibit the growth of reproductive testicular tumors by decreasing cell survival, proliferation, migration, and invasion 47. LncRNA *H19* knockdown in TCam-2/CDDP cells can help tumor cells survive by affecting cell activity reduction, cell cycle arrest, increase apoptosis, and decrease invasion 48. However, whether other lncRNAs also affect the tumorigenesis and progression of TGCTs was completely unknown. In this study, our results about the expression of *CSNK1G2-AS1* were consistent with the analysis from the GEPIA database described previously. Comparing with the normal testis, the testicular germ cell tumor tissues exhibited significantly decreased *CSNK1G2-AS1* expression, suggesting the potential involvement of *CSNK1G2-AS1* in the tumorigenesis and development of testicular tumors. Meanwhile, in TGCTs, low *CSNK1G2-AS1* methylation indicated high DFS, indicating the association of *CSNK1G2-AS1* with the prognosis of TGCT, while its role in TGCTs remains unclear. Therefore, we conducted an *in vitro* study to overexpress *CSNK1G2-AS1* in TCam-2 and NCCIT cells to investigate the role of *CSNK1G2-AS1* in TGCT cells. Through *in vitro* experiments, we found that overexpression of *CSNK1G2-AS1* did not affect the proliferation of TGCT cells but promoted clonal formation, invasion, and migration, as well as inhibited the apoptosis of TCam-2 and NCCIT cells. Therefore, *CSNK1G2-AS1* could promote the development of TGCTs by increasing the metastasis and survival rate of TGCT cells *in vitro*. Next, we explored the signaling pathway which involved in the promotion of *CSNK1G2-AS1* on the development of TGCTs.

Metastasis is an important reason for the occurrence, development, and poor prognosis of tumors 49. The metastasis and recurrence of TGCTs are some of the most intractable problems in a clinical setting. We found that *CSNK1G2-AS1* could promote TGCT cells migration and invasion, which further proved that *CSK1G2-AS1* can promote TGCTs development. The EMT is a developmental process that enables stationary epithelial cells to gain the ability to migrate and invade independent cells 50 and is associated with various tumor functions, including tumor initiation, malignant progression, tumor stem cell formation, tumor cell migration, infiltration into the blood, metastasis, and therapeutic resistance 51. An increasing amount of recent studies have shown that tumor metastasis may be significantly related to the EMT signaling pathway. For example, Lei *et al*. discovered that overexpression of miR-145 could reverse the EMT phenotype of thyroid cancer cells and reduce the efficiency of cell metastasis 52. Li *et al*. indicated that Emodin inhibits EMT and invasion of pancreatic cancer by upregulating microRNA-1271 53. In this study, we detected proteins related to the EMT signaling pathway and found that after overexpression of *CSNK1G2-AS1*, the expression of ZO-1 decreased. Studies have shown that CAPS1 could promote CRC cell metastasis *in vitro* by inducing EMT, including reduced expression of the epithelial markers E-cadherin and ZO-1 54. Zhang *et al*. indicated that decreased expression of ZO-1 is related to the metastasis of liver cancer 55. Therefore, *CSNK1G2-AS1* may promote TGCT cells migration and invasion by regulating the EMT signaling pathway.

Many cellular processes, including cell growth, survival, apoptosis, angiogenesis, metabolism, and protein synthesis, can be regulated by AKT 56. Numerous studies have shown that AKT expression is increased in most cancers 57. In the cancers with increased AKT expression, cytoplasmic P21 expression was also elevated, contributing to tumor progression, chemotherapeutic resistance, and poor prognosis 58. AKT phosphorylation leads to the activation of several substrates, such as BAD, caspase, and forkhead transcription factors, which could impair apoptosis and enhance survival 59. In this study, we found that the expression and the phosphorylation of AKT significantly increased after the overexpression of *CSNK1G2-AS1* in TGCT cells. Therefore, *CSNK1G2-AS1* may inhibit TGCT cells apoptosis through the AKT signaling pathway. Meanwhile, studies have shown that activation of AKT could promote the EMT phenotype of seminoma and enhance seminoma invasion 38. These results suggested that *CSNK1G2-AS1* may promote the development of TGCT cells through regulating the EMT and AKT related signaling pathways.

Our *in vitro* experiments showed the role and the possible mechanism of *CSNK1G2-AS1* in promoting TGCT cells development. However, the down-regulation of *CSNK1G2-AS1* in TGCTs and the correlation between *CSNK1G2-AS1* and the prognosis of TGCT patients suggested that *CSNK1G2-AS1* may resemble a tumor suppressor gene in TGCTs, contrary to the results of the cell experiments *in vitro*. From an objective point of view, the results of *in vitro* studies may not accurately represent the situation *in vivo*. Cancer development and progression are closely related to changes of the surrounding stroma. Cancer cells can functionally shape their microenvironment by secreting various cytokines, chemokines, and other factors 60. Tumor growth, metastasis, and response to treatment are also associated with the complexity of the tumor microenvironment (TME), which is an “ecological niche” that stimulates cancer progression. The dynamic changes that occur in TME could cause selection of tumor cell variants, which may promote genomic instability 61-63. The *in vitro* experiments of tumor cells lose the signals maintained or assisted *in vivo*, and lose contact with other cells or molecules, which may lead to inconsistent experimental results between *in vitro* and *in vivo* experiments. For example, arsenic trioxide (ATO) has opposite effects on the nuclear factor erythroid-derived 2 like 2 (Nrf2) pathway in oral squamous cell carcinoma *in vitro* and *in vivo* 64. Furthermore, the role of RASSF5 in cancer is controversial, Liao *et al*. explained how RASSF5 can activate MST1/2 and suppress cancer *in vivo* but inhibit MST1/2 *in vitro* 65. However, there is no suitable *in vivo* model for human TGCTs and no animal model capable of forming precursor cell carcinomas *in situ* observed in humans 66.

TGCTs originating from CIS can traditionally be divided into seminoma and non-seminoma, each accounting for approximately 50% 67. Seminoma consists of transformed germ cells that closely resemble primordial germ cells (PGCs)/gonocyte, and NSE can be composed of cells with typical pluripotency of PGCs/gonocytes 68. Therefore, the gene expression profile of TGCTs is similar to PGCs/gonocytes 69. Due to the limitations of sample acquisition, in the preliminary study of the expression characteristics of lncRNAs in TGCTs, the control group used was paracancerous testicular tissue. Spermatogenesis is a complex and dynamic cellular differentiation process. We found that in the spermatogenesis process, *CSNK1G2-AS1* expression level was high in the later stages of spermatogenesis but low in the proliferative stage of spermatogonial stem cells and meiophase. Perhaps PGCs/gonocytes would be a more appropriate control for the study of genetic/epigenetic lesions in TGCTs. The expression level of *CSNK1G2-AS1* in TGCTs compared to PGCs/gonocytes was still unknown, whether *CSNK1G2-AS1* was expressed at the wrong developmental node to promote the tumorigenesis and metastasis of TGCTs needs further investigation.

This study is the first to confirm the role of *CSNK1G2-AS1* in tumors and its role in TGCTs. Although we have studied the effect of *CSNK1G2-AS1* on TGCT cells, this study also has some limitations. Firstly, due to the difficulty for the collection of patient samples, the sample size of this study was not very sufficient. Secondly, we should further study how *CSNK1G2-AS1* affects the AKT and EMT signaling pathways and how the AKT and EMT signaling pathways affect the development of TGCT cells. Finally, we must determine the target genes of *CSNK1G2-AS1* in TGCTs to further study how they affect the tumorigenesis and prognosis of TGCT. The current findings suggest that *CSNK1G2-AS1* can be used as a biomarker for testicular germ cell tumor cells. On the basis of improving relevant studies, it is expected to develop inhibitors of *CSNK1G2-AS1* to inhibit the migration of testicular germ tumor cells, and develop new therapeutic drugs to improve TGCT treatment.

## Conclusion

In summary, the role of *CSNK1G2-AS1* in TGCTs was described for the first time in this study, and its molecular mechanism was preliminarily discussed. *In vitro* studies have shown that *CSNK1G2-AS1* could suppress TGCT cells apoptosis but promote colony formation, migration, and invasion of TGCT cells through the AKT and EMT signaling pathways, suggesting that *CSNK1G2-AS1* may play a critical role in the development and metastasis of TGCT cells.

## Abbreviations

TGCTs: Testicular germ cell tumors
*CSNK1G2-AS1*: Casein kinase 1 gamma 2 antisense RNA 1
GEPIA: Gene Expression Profiling Interactive Analysis
ANOVA: One-way analysis of variance
AUC: Area Under Curves
EMT: Epithelial-mesenchymal transformation
PGCs: Primordial germ cells
DFS: Disease-free survival
PFS: Progression-free survival
ROC: Receiver operating characteristic
MTS: 3-(4,5-dimethylthiazol-2-yl)-5-(3-carboxymethoxyphenyl)-2-(4-sulfophenyl)-2H-tetrazolium
